# Female Reproductive Performance in the Mouse: Effect of Oral Melatonin

**DOI:** 10.3390/molecules23081845

**Published:** 2018-07-25

**Authors:** Xiaoxue Zhao, Dian Wang, Zhenzheng Wu, Bo Pan, Haoxuan Yang, Changjun Zeng, Ming Zhang, Guoshi Liu, Hongbing Han, Guangbin Zhou

**Affiliations:** 1 Farm Animal Genetic Resources Exploration and Innovation Key Laboratory of Sichuan Province, College of Animal Science and Technology, Sichuan Agricultural University, Chengdu 611130, China; zxxzhaoxiaoxue@126.com (X.Z.); wangdian413@gmail.com (D.W.); WZZ15680826096@163.com (Z.W.); bopan1992@163.com (B.P.); yanghaoxuan940712@gmail.com (H.Y.); zengchj@sicau.edu.cn (C.Z.); zhangming@sicau.edu.cn (M.Z.); 2 National Engineering Laboratory for Animal Breeding, Key Laboratory of Animal Genetics and Breeding of the Ministry of Agriculture, Beijing Key Laboratory for Animal Genetic Improvement, College of Animal Science and Technology, China Agricultural University, Beijing 100193, China; gshliu@cau.edu.cn

**Keywords:** oral melatonin, mouse, litter size, follicular density, in vitro development, apopotosis-related genes

## Abstract

Although melatonin has some of the broadest ranges of actions on the physiology of vertebrates, especially on their reproductive processes, the mechanism by which melatonin regulates animal reproduction is still incompletely understood. This study was designed to determine the effect of oral melatonin on the reproductive performance of female mice. Female ICR mice (7 weeks old) were given melatonin-containing water (3, 30 and 300 μg/mL; melatonin) or water only (control) until 10 weeks of age. Then, some of the mice were successfully mated (confirmed by vaginal plugs), and the number of live births and their weights were recorded. Some mice were used for a histological analysis of the number of follicles in the ovaries. Others were used for oocyte collection after superovulation, and in vitro fertilization (IVF) was performed. The mRNA expression of the apopotosis-related genes (*BAX*, *BCL*2) in the IVF embryos were analyzed. After melatonin administration, the mice showed similar serum melatonin levels to that of the control. The number of antral follicles per mm^2^ unit area in the 30 μg/mL melatonin-treated group (14.60) was significantly higher than that of the control (7.78), which was lower than that of the 3 μg/mL melatonin-treated group (12.29). The litter size was significantly higher in the 3 μg/mL melatonin-treated group (15.5) than in the control (14.3). After IVF, the hatched blastocyst formation rate in the 30 μg/mL melatonin-treated group (85.70%) was significantly higher than that of the control (72.10%), and it was the same for the *BCL*2/*BAX* expression ratio. Although oral melatonin did not appear to have an effect on the serum melatonin rhythm in the mouse, melatonin did increase litter size at the 3 μg/mL dose level, and improved the developmental competency of IVF embryos at the 30 μg/mL level.

## 1. Introduction

Melatonin (MT, *N*-acetyl-5 methoxytryptamine), a hormone primarily secreted by the pineal gland, was first isolated and characterized from the bovine pineal gland by Lerner et al. in 1958 [1]. It was found that MT could regulate the seasonal reproduction of mammals through the hypothalamus-pituitary-gonadal axis [2]. In response to different day length, gonadotrophin-releasing hormone (GnRH) pulse generation by the hypothalamus varied [2], which was mainly resulted from changes in the nightly secretion of MT from the pineal gland with the changes of photoperiod, thus decreasing the negative feedback sensitivity of GnRH neurons to estradiol [3]. Reproductive functions may also be regulated by MT via direct action on the gonads. There are two subtypes of receptor, MT receptor 1 (MT1) and MT receptor 2 (MT2), in human luteal granulosa cells [4] and ovarian cells. Therefore, MT could act directly on the target organ (ovary) through its receptors and regulate the synthesis and secretion of gonadal hormones, thereby affecting reproductive functions [5,6]. Since fertility in mammals is a complex process dependent upon the successful completion of a number of critical steps from oogenesis, transport of spermatozoa within the female tract, capacitation and fertilization, early embryogenesis, maternal recognition of pregnancy, gestation and parturition, the mechanism by which melatonin regulates animal reproduction is still incompletely understood.

As the biological function of melatonin have been linked to circadian rhythms, endocrine functions and animal reproduction in general, the use of MT has attracted much attention both in basic research and commercial applications [7,8]. Oral MT could improve the quality of oocytes in aged ICR mice [9], the age-induced fertility decline in Kunming female mice [10] as well as oocyte quality in women undergoing in vitro fertilization-embryo transfer [11]. When receiving melatonin tablets (1–3 mg/day) for 6 weeks, patients with premature ovarian failure showed a higher percentage of normal excretion of ovarian hormones than controls in a one-year follow-up study [12]. When MT was added to the culture medium, it could promote blastocyst formation in mice [13], goats [14], cattle [15] and pigs [16]. Among these studies, the rate of blastocyst formation from activated porcine oocytes was increased from 18% to 28% with a final concentration of 10^−9^ M melatonin [16]. Whereas, another study showed that 10^−10^ M (MT) was the optimal concentration, which resulted in significantly increased parthenogenetic blastocyst formation rates (30.0% vs. 19.2%) in pigs [17]. While in cattle production, MT administration (4.64 mg per cow) significantly improved their first-cycle conception rate (44.4% vs. 35.9%) [18]. When MT (40 mg/animal) was subcutaneously implanted into deer before the breeding season, the average number of corpora lutea per animal was increased significantly from 5.75 to 9.75 [19]. MT implants also tended to improve the viability of embryos (67% vs. 43%) collected from aged ewes after superovulation [20], and the pregnancy rate (26.3% vs. 76.5%) of undernourished post-partum ewes [21] in the seasonal anestrous period.

Based on the previous results mentioned above, it seems that effects of MT on the developmental potential of oocytes or/and embryos can differ considerably from the types of MT administrations (oral, supplementation of culture medium and subcutaneous implantation). Even with culture medium supplementation, the optimal MT concentration varied among different laboratories [17,18]. Therefore, the current study was designed to evaluate the effects of exogenous melatonin on female ICR mice, specifically: (1) the serum melatonin rhythm; (2) the number of follicles in the ovaries and the average litter size of female mice after successful mating; and (3) the in vitro development of oocytes after in vitro fertilization (IVF) and the mRNA expression of the apopotosis-related genes (*BAX*, *BCL*2) in the IVF embryos.

## 2. Results

### 2.1. Effect of Melatonin on the Serum Melatonin Levels

**Figure 1:**
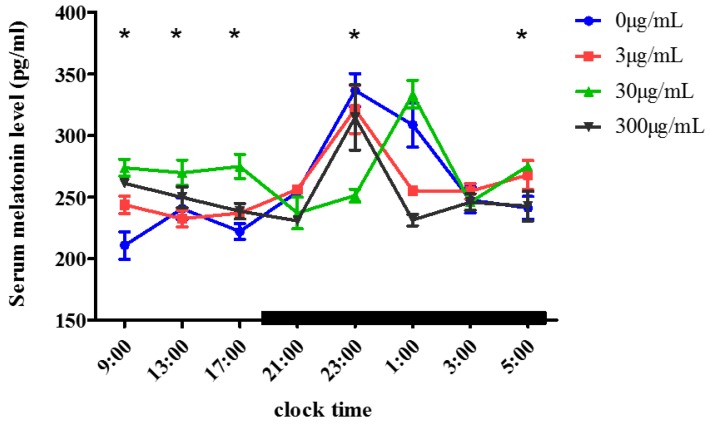
Serum melatonin levels in mice after oral melatonin. The mice were treated with different concentrations of melatonin (0 μg/mL, 3 μg/mL, 30 μg/mL, 300 μg/mL) in their drinking water for 3 weeks, then the serum melatonin was measured by ELISA at different time points (mean ± SD, *n* = 3). Bars indicate scotophase duration. “*” indicates that there was a significant difference (*p* < 0.05) between the 30 μg/mL melatonin-treated group and the control group at different time points.

We measured the water consumption when the female mice were aged from 5 to 8 weeks old, and found that the average water consumption for one mouse is about 3 mL per day. As shown in Figure 1, the serum MT levels in the melatonin-treated and control groups showed similar circadian rhythms: Low (210.64−236.96 pg/mL) in the daytime (9:00–17:00) and high (314.60–336.63 pg/mL) at night (21:00–5:00). In the 3 μg/mL and 300 μg/mL melatonin-treated groups, the concentration of serum MT began to increase at 21:00 (256.26 ± 0.22 pg/mL and 230.75 ± 4.94 pg/mL respectively), and peaked at 23:00 (321.25 ± 33.99 pg/mL; 314.59 ± 45.69 pg/mL), then decreased to daytime levels at 5:00 (268.00 ± 20.42 pg/mL; 242.59 ± 20.85 pg/mL). While in the 30 μg/mL melatonin-treated group, the serum MT level peaked at 1:00 (333.45 ± 19.74 pg/mL), was significantly higher (*p* < 0.05) in daytime (9:00–17:00) than that of the control but showed no significant difference from that of the control at night (1:00–3:00).

### 2.2. Effect of Melatonin on Litter Size and Birth Weight of Pups

**Figure 2:**
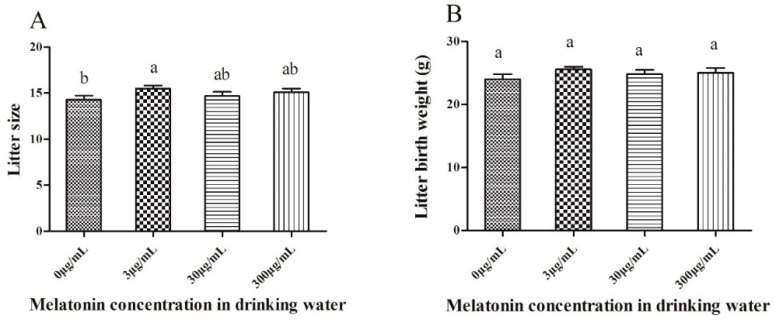
Effect of oral melatonin on litter size (**A**) and litter birth weights (**B**). Each value represents the mean ± SD from 10 replicates performed repeatedly. (a, b) indicate significant differences between the different groups (*p* < 0.05).

As shown in Figure 2A, the litter size was significantly higher (*p* < 0.05) in the 3 μg/mL melatonin-treated group (15.5 ± 1.08) than that of the control (14.3 ± 1.42), which was similar to that of the other two melatonin-treated groups (30 µg/mL: 14.7 ± 1.42; 300 µg/mL: 15.1 ± 1.29). Also, the average total weight of the litter was higher in the melatonin-treated groups (24.83 to 25.57 g) than in the control (24.01); however, there was no significant difference between them (Figure 2B). Moreover, there is no significant difference in mean pup weight between groups (data not shown).

### 2.3. Effect of Melatonin on Follicular Development in Mouse Ovaries

**Figure 3:**
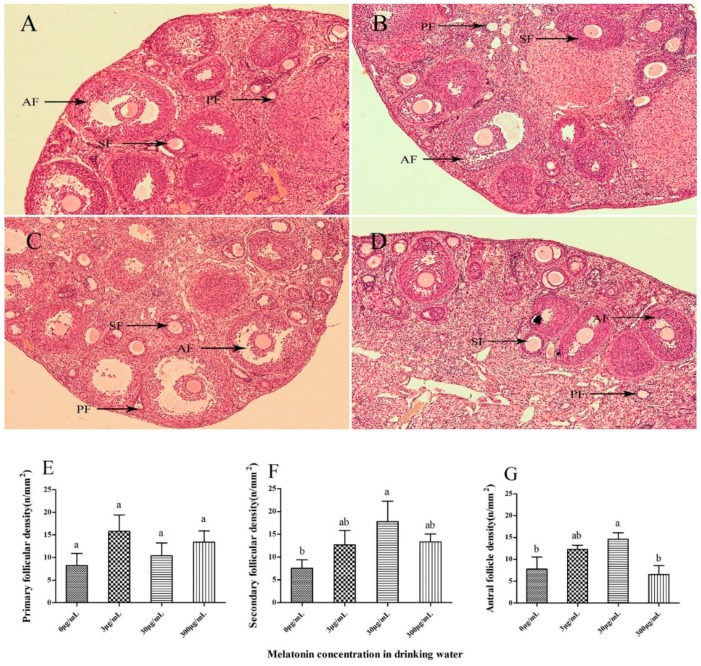
Light microscopy images of mouse ovarian tissues. The tissues displaying a morphologically normal primary follicle (PF) and a secondary follicle (SF) stained with hematoxylin and eosin composed of intact oocytes and well-organized granulosa cells and the antral follicle (AF) were morphologically normal in the control group (**A**), the 3 μg/mL melatonin-treated group (**B**), the 30 μg/mL melatonin-treated group (**C**) and the 3 μg/mL melatonin-treated group (**D**). primary follicular density (**E**), secondary follicular density (**F**) and antral follicular density (**G**) among all groups. Original magnification 100×. The number of follicles at various developmental stages per unit area (mm^2^) of the ovarian cortex, follicular density (n/mm^2^), was counted. Different superscripts (a and b) represent treatment differences within panels (*p* < 0.05).

The typical morphology in the mouse ovary as visualized after hematoxylin–eosin (HE) staining is shown in Figure 3A–D. Morphological evaluation was performed to compare the structures of primary, secondary and antral follicles in mouse ovarian sections from all groups. During histological analysis, the integrity of the ovaries was well preserved in all melatonin-treated groups, which were similar to that of control. Morphologically normal follicles were characterized by a round or oval oocyte, presenting a well-delimited nucleus with uncondensed chromatin, surrounded by healthy granulosa cells closely juxtaposed to the oocyte. The number of secondary follicles (17.78 ± 8.99/mm^2^, Figure 3F) and antral follicles (14.60 ± 2.92 cells/mm^2^, Figure 3G) in the 30 μg/mL melatonin-treated group were significantly higher than in the control group (7.54 ± 3.76 and 7.78 ± 5.52 cells/mm^2^, respectively) (*p* < 0.05) in cortical area, but there was no significant difference in primary follicular density among all groups (Figure 3E).

### 2.4. Effect of Melatonin on Blastocyst Formation

As shown in Table 1, there was no significant difference in the percentage of IVF embryos developed to the 2-cell stage (87.5 to 95.8%) among all the melatonin-treated groups. When IVF embryos developed into expanded blastocysts, their percentage was significantly lower (*p* < 0.05) in the 300 μg/mL melatonin-treated group (63.62%) than that of the other melatonin-treated groups (87.10 to 87.73%) and the control group (81.11%). However, the rate of hatched blastocysts was significantly higher (*p* < 0.05) in the 30 μg/mL melatonin-treated group (85.70%) than in the control (72.10%).

### 2.5. Effect of Melatonin on Expression of Apoptosis-Related Genes in IVF-Derived Mouse Embryos

**Figure 4:**
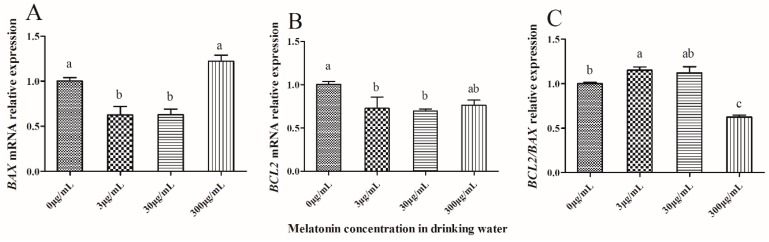
Effect of melatonin on gene expression of mRNA in mouse IVF-derived 2-cell embryos. *BAX* expression (**A**), *BCL*2 expression (**B**) and *BCL2/BAX* relative expression (**C**) among all groups. The relative expression levels of mRNA were determined by the 2^−ΔΔCT^ method and normalized against GAPDH. All data are the mean ± SD from 3 replicates. Different superscripts (a, b) represent treatment differences within panels (*p* < 0.05).

As shown in Figure 4, the expression of *BAX* and *BCL*2 in IVF-derived 2-cell embryos was significantly lower (*p* < 0.05) in the 3 μg/mL and 30 μg/mL melatonin-treated groups than that of the control group (*p* < 0.05) (Figure 4A,B), but the ratio of *BCL*2/*BAX* was significantly higher (*p* < 0.05) in the 3 μg/mL melatonin-treated group than that of the control, which was significantly higher (*p* < 0.05) than that of the 300 μg/mL melatonin-treated group (Figure 4C).

**Figure 5:**
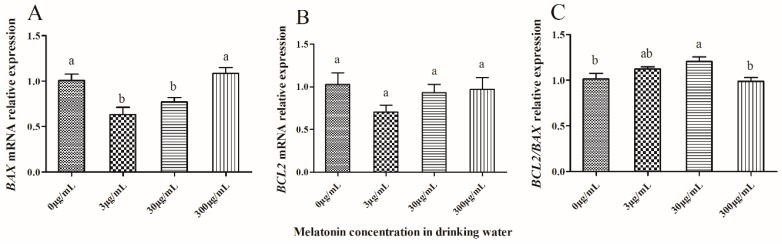
Effect of melatonin on gene expression of mRNA in mouse IVF-derived hatched blastocysts. *BAX* expression (**A**), *BCL*2 expression (**B**) and *BCL2/BAX* relative expression (**C**) among all groups. The relative expression levels of mRNA were determined by the 2^−ΔΔCT^ method and normalized against GAPDH. All data are the mean ± SD from 3 replicates. Different superscripts (a, b) represent treatment differences within panels (*p* < 0.05).

When the IVF-derived 2-cell embryos developed in vitro to the hatched blastocyst stage, the expression of *BAX* was significantly lower (*p* < 0.05) in the 3 μg/mL and 30 μg/mL melatonin-treated groups than that of the control (Figure 5A), and the *BCL*2 expression level showed no significant difference (*p* > 0.05) among all four groups (Figure 5B). The ratio of *BCL*2/*BAX* was significantly higher (*p* < 0.05) in the 30 μg/mL melatonin-treated group than that of the control, which was similar to that of 300 μg/mL melatonin-treated group (Figure 5C).

## 3. Discussion

In the present study, when pubertal female mice were treated with different concentrations (0–300 μg/mL) of MT in their drinking water for 3 weeks, the serum MT levels were low in the daytime and high at night, and the circadian rhythms were similar to those of a previous report that serum MT levels were low in the daytime and high at night before and after oral administration of three gelatin capsules, each containing 80 mg crystalline MT at 60 min intervals [22]. Although the serum MT levels were different between our study and the previous report [22] in the daytime and at night, the peak values were observed at night (314.59–336.63 pg/mL vs. about 100,000 pg/mL). The difference in serum MT levels may result from the different species studied (mouse vs. human). Oral melatonin is rapidly absorbed in humans; its elimination is more rapid than in sheep, but slower than in rodents [22]. Therefore, the large amount of MT synthesized by the pineal at night [23] and the exogenous MT could not accumulate in the blood [24], potentially resulting in the similar serum melatonin levels between groups at night and the similar circadian rhythms of melatonin in the present study. Furthermore, it would be expected that mice would consume more water in the night (being nocturnal), leading to a greater effect at night. Although the circadian rhythms of MT did not change after oral administration, the serum MT levels in the 30 μg/mL melatonin-treated group in our study peaked at 1:00, 2 h later than the normal time of 23:00. Further study should be instituted into the underlying mechanism of this phenomenon.

Accurate estimation of the number of ovarian follicles at various stages of development is an important indicator of the process of folliculogenesis [25]. In the present study, after oral MT at a dose of 30 μg/mL in the drinking water, the mice exhibited a higher number of antral follicles per area (mm^2^) of ovarian structures (14.60) than the control (7.78) and the other melatonin-treated groups (6.52 to 12.99), suggesting that MT at a concentration of 30 μg/mL benefits follicular development. Melatonin is a highly lipophilic molecule that crosses cell membranes to easily reach subcellular compartments including mitochondria [26], and could be efficiently absorbed in the ovary. Then, the high concentration of melatonin in the follicular fluid could act as a modulator of ovarian function through its receptors in granulosa-luteal cells [4]. When the concentration of exogenous MT is lower or higher than 30 μg/mL, it might negatively affect the MT level in follicular fluid, potentially leading to lower binding of MT to its receptors and the reduced number of antral follicles observed.

In this study with mice, the IVF-derived hatched blastocyst rate in the 30 μg/mL melatonin-treated group (85.7%) was significantly higher than that of the control (72.1%), and was the highest among all the melatonin-treated groups (3 μg/mL, 30 μg/mL and 300 μg/mL). In a previous report, MT increased the fertilization rate significantly at a concentration between 10^−6^ and 10^−4^ M (27.6 vs. 43.9 or 40.4%), however, a significant increase in the rate of mouse embryos reaching blastulation (8.9 vs. 23.5%) was only observed when they were cultured in a medium containing 10^−6^ M melatonin [27]. Although the route of MT addition is different (oral vs. cell culture) in both studies, the results were almost the same which demonstrated that exogenous MT could improve the in vitro development of IVF embryos at an optimal concentration, especially at lower levels. When mouse embryos were cultured in vitro, the generation of reactive oxygen species (ROS) was increased [28], but the excessive ROS could be eliminated by exogenous MT, potentially promoting IVF of oocytes and their subsequent development [11,29]. When the concentration of exogenous MT was above the optimal level (here perhaps 30 μg/mL), more and more ROS could be scavenged and the balance between the oxidation–reduction reactions and the intracellular antioxidative system would be influenced, significantly reducing cell viability [30].

In response to ROS, the functional balance of pro-apoptotic (BAX, BAK, BAD) and anti-apoptotic (*BCL*2, *BCL*-w, *BCL*-xl) mitochondrial proteins is altered which eventually leads to apoptosis [31,32]. That is why we measured the *BCL*2*/BAX* ratio in the IVF embryos at different stages and tried to investigate the effect of oral MT on in vitro development of IVF embryos. Here, the *BCL*2*/BAX* ratio in the derived hatched blastocysts was significantly higher in the 30 μg/mL melatonin-treated group than in the control. Cell death is a widespread feature in the blastocysts of many mammals and is regulated by the activity of apoptosis genes [33]. Overexpressed *BAX* counters the death repressor activity of *BCL*2 and accelerates apoptotic death, which suggests that the ratio of *BCL*2*/BAX* determines survival or death following an apoptotic stimulus [34]. It seems that in the appropriate range the higher the ratio of *BCL*2*/BAX* in the cell, the less cell death appears, inevitably leading to improved development of embryos.

## 4. Materials and Methods

Unless otherwise stated, all reagents were purchased from Sigma-Aldrich (St. Louis, MO, USA). All animals were maintained and handled in accordance with the requirements of the Institutional Animal Care and Use Committee of the Sichuan Agricultural University.

### 4.1. Animals and Drinking Water

Outbred female ICR mice aged 5 weeks were purchased from Dashuo Company (Chengdu, China). Then they were kept in autoclaved cages (4 mice per cage) in a room under standard conditions of 14:10 light/dark cycle (light on at 06:00 a.m.) and temperature (22 °C ± 2 °C). After two weeks of acclimation, the mice (body weight 26.05 ± 0.77 g) were treated with different concentrations of melatonin in drinking water (0, 3, 30 and 300 μg/mL) for 21 days. They had free access to water and food. The stock solution of melatonin was prepared in 98% ethanol, with a final ethanol concentration in the drinking water of 0.12%. Vehicle-treated controls received 0.12% ethanol in drinking water. Bottles containing drinking water were protected from light by tin foil and the water was changed every other day.

### 4.2. Serum Melatonin Assay

After 21 days, blood was collected from groups of four mice via the eyelid venous plexus at eight different time intervals (09:00, 13:00, 17:00, 21:00, 23:00, 01:00, 03:00 and 05:00), then they were mixed into a blood cell sample followed by centrifugation at 2000 r/min for 15 min to collect serum for the detection of MT content. Three serum samples collected from 12 mice in 3 fixed cages (4 mice per cage) represented 3 replicates. At night, the animals’ blood was collected under dim red light. Serum samples were kept frozen at −20 °C until assayed. The detection procedures followed the instructions for an enzyme-linked immunosorbent assay (ELISA) kit (DRE-M9079c, kmaels (Shanghai) Biotechnology Co., Ltd., Shanghai, China). The intra- and inter-assay coefficients of variation were 7–8%. Sensitivity of the assay was 10 pg/mL.

### 4.3. HE Staining and Histological Analysis

Haematoxylin and eosin (HE) staining was performed based on a previous description [35] with some modification. Mouse ovarian tissues were fixed in 4% paraformaldehyde for 24 h and then dehydrated in an alcohol gradient. The fixed tissues were embedded in paraffin wax. Serial sections 4 μm thick were prepared and every 11th section of each tissue piece was mounted on glass slides and stained with hematoxylin for 3–8 min followed by eosin dye solution for 1–3 min. Then the stained slices were removed after dehydration and transparency in a gradient of alcohol and xylene, and then sealed with neutral gum. The number of follicles at different developmental stages was classified according to a previous report [36]: (i) primary follicles: oocytes surrounded by a complete, single layer of cuboidal granulosa cells; (ii) secondary follicles: oocytes surrounded by two or more layers of cuboidal granulosa cells; and (iii) antral follicles: marked by the formation of a fluid-filled cavity adjacent to the oocyte called the antrum. To prevent double counting, each follicle was followed through neighbouring sections and counted only once. The follicular density, which was defined as the number of follicles at various developmental stages per unit area (mm^2^) of the ovarian cortex, was calculated and used as a statistical indicator.

### 4.4. Litter Size and Litter Birth Weights

After administration of MT for 21 days, a female mouse showing natural estrus was placed into the same cage with a male at 19:00 and separated the next morning (Day 0.5). Then, this process was repeated for the second, third and fourth nights. Successful mating was confirmed by the presence of a vaginal plug. The total number of female mice with vaginal plugs was 40, which represented 10 mice for each group. Nineteen days after successful mating, the number of newborns delivered from the pregnant females and their weights were recorded.

### 4.5. In Vitro Fertilization (IVF) and In Vitro Embryo Culture

After three weeks of melatonin administration, female mice were induced to superovulate by an intraperitoneal injection of 10 IU equine chorionic gonadotropin initially, and 48 h later, 10 IU human chorionic gonadotropin (hCG) was injected to trigger ovulation. Cumulus-oocyte complexes were collected from oviducts at 12–14 h after hCG treatment and recovered in M2 medium supplemented with 3 mg/mL bovine serum albumin. Cumulus cells were transferred to human tubal fluid (HTF) medium and then washed in M2 three times for experimental use. Then, 10 μL of capacitated spermatozoa, which had been incubated for 1–1.5 h in HTF medium in a CO_2_ incubator, was added to the oocytes. The final concentration was 2.0–6.0 × 10^6^ sperm/mL. Five h after insemination, the oocytes were removed from the fertilization drops, washed in KSOM-AA medium (CAT# MR-121-D, Millipore, Burlington, MA, USA) 3 times, and cultured in 70 μL drops of KSOM-AA medium (Millipore, Burlington, MA, USA). Embryos at the 2-cell, 4-cell, early blastocyst, expanded and hatched blastocyst stages were examined and recorded at 24, 48, 72, 108 and 120 h after insemination, respectively.

### 4.6. Quantitative Polymerase Chain Reaction (Q-PCR)

Total RNA was isolated from IVF embryos at the 2-cell (*n* = 30) and hatched blastocyst (*n* = 6) stages using Trizol reagent (Invitrogen, Carlsbad, CA, USA), respectively. The RNA was reverse transcribed into complementary DNA (cDNA) using the High Capacity cDNA Reverse Transcription (RT) kit (Applied Biosystems, Foster, CA, USA); then the cDNA was quantified by Q-PCR using a SYBR PrimeScript RT-PCR Kit (TaKaRa, Dalian, China) on a CFX96 Real-Time PCR Detection System (Bio-Rad, Hercules, CA, USA) under standard conditions. The cycle threshold (Ct) value used to calculate the relative expression levels was the average of three replicates and was normalized against that of the reference gene (GAPDH). The primer information is summarized in Table 2. The mRNA expression levels were calculated using the 2^−∆∆Ct^ method [37].

### 4.7. Statistical Analysis

Statistical analysis was conducted by one-way ANOVA followed by the LSD test using SPSS statistical software (IBM, Armonk, NY, USA). Data were expressed as the mean ± standard error, and *p* < 0.05 was considered statistically significant. Experiments were repeated at least 3 times.

## 5. Conclusions

To summarize, oral administration of melatonin to mice at a concentration between 3 and 300 μg/mL in the drinking water for 3 weeks did not change their serum melatonin rhythm, but the 30 μg/mL dose of melatonin did increase the serum melatonin level in the daytime (9:00–17:00), the number of antral follicles in the ovary, and the ratio of *BCL*2/BAX in IVF-derived hatched blastocysts, potentially contributing to the increased developmental potential of IVF mouse embryos.

## Figures and Tables

**Table 1:** Effect of oral MT on embryo development of mouse metaphase II oocytes after IVF.

Group (Oral MT)	No. of Oocytes Used for IVF	No. of Oocytes Developed to				
		2-Cell Embryos (%)	4-Cell Embryos (%)	Early Blastocyst (%)	Expanded Blastocyst (%)	Hatched Blastocyst (%)
0 μg/mL	146	137(93.48 ± 5.70)	133(90.06 ± 3.02) ^ab^	127(86.23 ± 4.74) ^a^	119(81.11 ± 4.46) ^a^	107(72.10 ± 8.13) ^bc^
3 μg/mL	152	145(95.78 ± 2.78)	143(93.35 ± 3.23) ^a^	138(91.08 ± 3.19) ^a^	135(87.73 ± 3.88) ^a^	131(83.89 ± 6.46) ^ab^
30 μg/mL	180	170(95.38 ± 5.81)	169(94.65 ± 5.38) ^a^	158(88.51 ± 7.82) ^a^	155(87.10 ± 8.89) ^a^	152(85.70 ± 8.10) ^a^
300 μg/mL	110	94(87.5 ± 12.8)	87(82.80 ± 15.18) ^b^	72(67.66 ± 14.34) ^b^	68(63.62 ± 12.83) ^b^	64(60.32 ± 17.24) ^c^

MT: melatonin; IVF: in vitro fertilization. Percentage data are presented as the mean ± SD from at least 3 replicates. Values with different superscripts (a, b and c) within each column differ significantly (*p* < 0.05).

**Table 2:** PCR primers used for SYBR green Q-PCR analysis.

Gene	Assay ID	Primer Seq (5′–3′)	Product Length	Tm (°C)
*BCL*2	NM_009741.5	F: AGGATTGTGGCCTTCTTTGA	120	60
		R: CAGATGCCGGTTCAGGTACT		
*BAX*	NM_007527.3	F: TGGAGATGAACTGGACAGCA	117	60
		R: TGAAGTTGCCATCAGCAAAC		
*GAPDH*	NM_001289726.1	F: AGAACATCATCCCTGCATCC	124	63
		R: AGATCCACGACGGACACATT		

## Abbreviations

GnRH	gonadotropin-releasing hormone
hCG	human chorionic gonadotropin
HE	hematoxylin and eosin
HTF	human tubal fluid
IVF	in vitro fertilization
MT	Melatonin
Q-PCR	Quantitative polymerase chain reaction
ROS	reactive oxygen species
